# The relationship between cognitive complaints and burden of non-cognitive symptoms in multiple sclerosis

**DOI:** 10.1192/bjo.2026.11041

**Published:** 2026-05-06

**Authors:** Stefanie Roberts, Valeriya Kuznetsova, Fiore D’Aprano, Carmen J. Zheng, Tomas Kalincik, Charles B. Malpas

**Affiliations:** Neuroimmunology Centre, Department of Neurology, The Royal Melbourne Hospital, Melbourne, Australia; Clinical Outcomes Research (CORe) Unit, Department of Medicine (RMH), The University of Melbourne, Melbourne, Australia; Centre of Excellence for Cellular Immunotherapy and Clinical Haematology, Peter MacCallum Cancer Centre and Royal Melbourne Hospital, Melbourne, Australia; Melbourne School of Psychological Sciences, The University of Melbourne, Parkville, Australia; Florey Department of Neuroscience and Mental Health, The University of Melbourne, Parkville, Australia

**Keywords:** Multiple sclerosis, cognition, fatigue, sleep, depression

## Abstract

**Background:**

Cognitive complaints are common in multiple sclerosis, but their relationship to non-cognitive symptoms such as fatigue, sleep dysfunction and psychopathology has not been systematically examined in patients referred for specialist cognitive evaluation. These potentially modifiable symptoms may warrant attention in a clinical context.

**Aims:**

This study aimed to characterise common patterns of cognitive and non-cognitive symptoms in a referred patient cohort and determine whether cognitive complaints are associated with clinically significant fatigue, sleep dysfunction and psychopathology.

**Method:**

Cognitive complaints were captured using (a) a binary classification derived from clinical impression and (b) a severity rating from a self-report instrument. Objective cognitive performance was measured across five cognitive domains. Patients also completed self-report measures of fatigue, sleep dysfunction and psychopathology.

**Results:**

Fifty-one patients were included. Although 98% had cognitive complaints, only 29% had objective cognitive impairment. Most (90%) had significant non-cognitive symptoms, primarily fatigue (86%), sleep dysfunction (28%) and depression (26%). Pattern analysis revealed that the most common symptom phenotype was cognitive complaints with significant non-cognitive symptoms, occurring in the absence of objective cognitive impairment. More severe cognitive complaints were associated with greater psychopathology (*r* = 0.57, *BF*_10_ = 2188.48), fatigue (*r* = 0.53, *BF*_10_ = 366.44) and sleep dysfunction (*r* = 0.47, *BF*_10_ = 69.27).

**Conclusions:**

Cognitive complaints in multiple sclerosis may reflect broader non-cognitive symptom burden rather than objective cognitive impairment, even among patients referred for specialist evaluation. Their presence should prompt consideration of fatigue, sleep disturbance and psychopathology as potential targets for intervention.

The management of cognitive function is a high priority for patients with multiple sclerosis, given its adverse impact on employment, social functioning and quality of life.^1–3^ Objective cognitive impairment (i.e. poor performance on cognitive tests) is a relatively common symptom of multiple sclerosis, affecting approximately 30% of patients with a relapsing-remitting disease course (RRMS).^4^ In contrast, up to 70% of patients with RRMS report some degree of subjective cognitive complaint (i.e. experience of cognitive difficulties, usually measured with patient-report rating scales; see Box 1).^5^

Box 1 Definitions of cognitive and non-cognitive terms

**Cognitive complaints:** Subjective experiences of cognitive difficulty reported by the patient (e.g. problems with memory, attention or word-finding), which may or may not correspond to objective cognitive impairment on standardised testing.

**Objective cognitive performance:** Continuous scores derived from standardised neuropsychological tests.

**Objective cognitive impairment:** Performance below expected levels on standardised neuropsychologist tests, defined using established normative thresholds.

**Cognitive domains:** Discrete components of cognitive function (e.g. attention and processing speed, executive function, memory, learning and language).

**Non-cognitive symptoms:** Clinically relevant subjective symptoms other than cognitive complaints, including fatigue, mood disturbance and sleep-related difficulties.

The relationship between objective cognitive impairment and cognitive complaints in multiple sclerosis remains unclear. A recent meta-analysis suggests weak convergence between the two, with cognitive complaints accounting for as little as 3% of the variance in cognitive test performance.^6^ Because of this discrepancy, clinical guidelines prioritise test performance as the most useful method for detecting cognitive dysfunction in clinical settings.^7,8^ Cognitive complaints may, however, be sensitive to factors beyond objective cognitive impairment that can affect cognitive performance. This pattern is not unique to multiple sclerosis; across broader clinical and mental health settings, cognitive complaints frequently show weak correspondence with objective cognitive performance, and are more strongly associated with internalising symptoms such as depression and anxiety.^9^ Fatigue and internalising symptoms are also consistently associated with greater cognitive complaints in the absence of objective cognitive impairment in patients with multiple sclerosis.^10–18^ Poor sleep has also been linked to negative perceptions of cognitive function in multiple sclerosis.^19,20^ These non-cognitive symptoms are clinically relevant, as they may represent modifiable targets for intervention.

To date, no studies have directly examined the clinical utility of cognitive complaints in a specialist cognitive clinic for patients with multiple sclerosis. Cognitive complaints are expected to be highly prevalent in this setting, as referrals are typically prompted by patient-reported difficulties or clinician concern. The prevalence of cognitive complaints in this context is therefore likely to differ from that observed in the general multiple sclerosis population. Despite this, the role of cognitive complaints in guiding clinical decision-making remains unclear. Prior research has primarily relied on patient-report rating scales to measure cognitive complaints, but this method may not fully align with how healthcare providers conceptualise concerns in practice. Anecdotally, multiple sclerosis healthcare providers often make a binary judgement about whether a patient expresses a cognitive complaint, a distinction which may carry different clinical implications than severity ratings alone. For example, the presence of a cognitive complaint may serve as the basis for a referral to clinical neuropsychology, influence the assessment of disease stability and overall disability, or be included in the differential diagnostic process. Prior studies of non-cognitive symptoms have also typically used brief screening measures of psychopathology restricted to depression and/or anxiety (e.g. the Hospital Anxiety and Depression Scale)^21^ and limited cognitive tests (e.g. the Brief International Cognitive Assessment for Multiple Sclerosis (BICAMS)),^22^ which may not capture the heterogeneity of multiple sclerosis-related cognitive impairment.^23^ A more comprehensive approach may be achieved by incorporating dimensional models of psychopathology that assess a broader range of symptom domains, along with a cognitive test battery designed to evaluate performance across multiple cognitive domains.

The primary aim of this study was to investigate the clinical significance of cognitive complaints in areas beyond objective cognitive impairment in patients with multiple sclerosis who were referred to a specialist cognitive clinic. Specifically, the study characterised the clinical profiles of referred patients by examining the distribution and co-occurrence of cognitive complaints, objective cognitive impairment and non-cognitive symptoms that may warrant clinical attention. A secondary aim was to compare different approaches to capturing cognitive complaints, including a clinician-derived binary classification and a validated self-report severity scale. It was hypothesised that cognitive complaints would be more closely associated with non-cognitive symptoms than with objective cognitive impairment, highlighting their potential relevance for broader clinical management.

## Method

### Setting

Data for this retrospective record review were obtained from patients referred to the Cognitive Neuroimmunology Clinic at The Royal Melbourne Hospital between March 2020 and October 2024. Under the clinical service model, patients could be referred for investigation on the basis of a cognitive complaint, a concern raised by the primary treating neurologist or for baseline assessment.

### Standard protocol approvals, registrations and patient consents

The authors assert that all procedures contributing to this work comply with the ethical standards of the relevant national and institutional committees on human experimentation and with the Helsinki Declaration of 1975, as revised in 2013. All procedures involving human patients were approved by The Melbourne Health Human Research Ethics Committee (reference number QA2021089). The committee waived the requirement for patient consent for the collection, analysis and publication of retrospectively obtained, anonymised data for this non-interventional study.

### Study population

Patients were included if they had a diagnosis of multiple sclerosis, were aged ≥18 years, were referred for specialist cognitive opinion during the study period and had complete data for the relevant variables. No exclusions were applied on the basis of comorbid psychiatric symptoms, fatigue, sleep disturbance or cognitive impairment. Clinical variables were collected from patient files, including gender, age, education, disease duration and Expanded Disability Status Scale (EDSS)^24^ score.

### Neuropsychological examination

All patients underwent a comprehensive neuropsychological assessment, including clinical history, targeted psychometric testing and tailored interventions when indicated. Given the retrospective nature of the study, information regarding the order of formal examination, time of day and standardisation of testing procedures was not consistently available from patient records. All assessments were, however, conducted as part of a single approximately 1.5 h consultation, of which approximately 60 min involved psychometric testing. Self-report questionnaire data were collected digitally and completed by patients in their own time. For this study, the presence or absence of cognitive complaints (true/false) was determined based on whether a complaint was elicited during the neuropsychological consultation. The classification was determined by the treating neuropsychologist immediately following the consultation, blind to self-report scale outcomes. The severity of the cognitive complaint was determined using a self-report scale, as described below.

Consistent with the International Classification of Cognitive Disorders in Multiple Sclerosis (IC-CoDiMS),^25^ a cognitive composite approach was used to identify objective cognitive impairment. Measures were selected for their reliability and coverage of distinct cognitive domains, including attention and processing speed, executive function, new learning, memory recall and language. Raw test scores were standardised to *z*-scores (mean 0, s.d. = 1) using age-, education- and/or gender-adjusted published normative data. Composite scores were calculated as the mean *z*-scores across tests within each cognitive domain. Consistent with the IC-CoDiMS methodology, objective cognitive impairment was defined as the presence of at least two subtests within a composite scoring at or below *z* = −1.5 s.d. The tests comprising each cognitive composite and associated normative reference are described in detail in Supplementary Table 1 available at https://doi.org/10.1192/bjo.2026.11041.

All patients completed the SPECTRA: Indices of Psychopathology (SPECTRA),^26^ a 96-item validated self-report questionnaire designed to capture psychopathology within a hierarchical-dimensional framework. Consistent with widely replicated models of psychopathology,^27^ the SPECTRA assesses 12 non-overlapping clinical scales that are organised into three higher-order spectra: internalising, externalising and reality-impairing. These domains contribute to a composite index known as the general psychopathology index (GPI), which reflects global psychological burden. The SPECTRA also includes three supplemental scales assessing psychosocial functioning, suicidal ideation and cognitive complaints.

The SPECTRA cognitive concerns subscale served as the primary measure of severity of cognitive complaints in all analyses. Severity of concerns was operationalised dimensionally, reflecting the degree to which patients endorsed cognitive difficulties on the self-report scale, rather than to predefined thresholds. This is in contrast to the binary definition of complaint that was also investigated in this study. Items probing severity of cognitive complaints include difficulties with word finding, planning of daily activities, personal organisation and prospective memory. The SPECTRA cognitive concerns subscale was selected because it was developed specifically for neuropsychological clinical and research applications and situates subjective cognitive complaints within a broader dimensional model of psychopathology. In this framework, cognitive complaints are conceptualised as a supplemental but theoretically integrated domain, rather than as a stand-alone or disorder-specific construct. This approach allowed cognitive complaints to be examined in relation to broader symptom dimensions, using a common measurement approach. Robustness of this measurement choice was evaluated through sensitivity analyses by using alternative multiple sclerosis-specific measures described below. In accordance with the test manual, *t*-scores (mean 50, s.d.= 10) were calculated for each scale, each spectra and the GPI. Higher *t*-scores indicate greater psychopathological burden. See Table 1 for symptom scales and higher-order indices.

**Table 1:** Patient characteristics and correlations with SPECTRA cognitive concerns

Characteristic	Mean (s.d.)	Pearson’s *r* [95% CI]	*BF*_10_
Clinical characteristic			
Age, years	43.31 (10.75)	−0.19 [−0.43 to 0.09]	0.43
Education, years	13.31 (2.27)	−0.08 [−0.34 to 0.20]	0.20^a^
Disease duration, years	9.79 (8.82)	−0.21 [−0.44 to 0.07]	0.48
EDSS	2.43 (1.91)	0.14 [−0.15 to 0.40]	0.28^a^
Cognitive domain			
Attention and processing speed	−0.12 (0.76)	−0.37 [−0.57 to −0.09]	5.06^b^
Executive function	−0.31 (0.60)	−0.21 [−0.44 to 0.07]	0.48
New learning	0.04 (1.09)	−0.19 [−0.43 to 0.09]	0.40
Memory recall	0.10 (0.99)	−0.21 [−0.45 to 0.07]	0.49
Language	−0.12 (0.81)	−0.20 [−0.48 to 0.03]	0.82
Non-cognitive symptom			
Sleep disturbance	56.21 (8.46)	0.20 [−0.08 to 0.44]	0.44
Sleep impairment	59.06 (9.74)	0.47 [0.22−0.65]	69.27^b^
Fatigue	78.14 (15.58)	0.53 [0.28−0.69]	366.44^b^
SPECTRA general psychopathology index	57.49 (9.84)	0.57 [0.49−0.80]	2188.48^b^
Internalising spectrum	62.86 (13.35)	0.54 [0.30−0.70]	602.57^b^
Depression	64.08 (14.45)	0.52 [0.27−0.68]	275.66^b^
Anxiety	61.18 (15.18)	0.43 [0.17−0.62]	24.07^b^
Social anxiety	58.78 (14.39)	0.49 [0.23−0.66]	96.54^b^
Post-traumatic stress	59.55 (14.21)	0.36 [0.08−0.56]	4.24^b^
Externalising spectrum	50.47 (11.24)	0.33 [0.05−0.54]	2.47
Alcohol problems	50.16 (19.80)	0.33 [0.52−0.54]	2.47
Severe aggression	47.24 (8.76)	0.22 [−0.06 to 0.46]	0.56
Antisocial	47.65 (8.92)	0.30 [0.03−0.52]	1.64
Drug problems	46.12 (7.06)	0.22 [−0.06 to 0.46]	0.57
Reality-impairing spectrum	46.14 (7.59)	0.28 [0.01−0.51]	1.23
Psychosis	46.80 (9.00)	0.02 [0.01−0.51]	0.18^a^
Paranoid ideation	46.78 (8.51)	0.18 [−0.25 to 0.28]	0.37
Manic activation	52.35 (10.13)	0.24 [−0.10 to 0.42]	0.68
Grandiose ideation	40.74 (7.87)	0.24 [−0.04 to 0.47]	0.69
Psychosocial functioning	45.29 (9.87)	−0.44 [−0.62 to −0.17]	24.33^b^
Suicidal ideation	51.55 (14.94)	0.28 [0.01−0.51]	1.24
Cognitive concerns	72.29 (16.83)	–	–

BF10, Bayes factor for the alternative hypothesis; EDSS, Expanded Disability Status Scale.

Cognitive terms represent *z*-scores. Sleep disturbance, sleep impairment, fatigue and SPECTRA terms represent *t*-scores.

a. Bayes factor <1/3.

b. Bayes factor >3.

Patients also completed the modified Fatigue Impact Scale.^28^ *t*-Scores were calculated based on previously published age-, gender- and education-adjusted normative data.^29^ Sleep quality was measured with the Patient Reported Outcomes Measurement Information System (PROMIS) computerised adaptive tests for Sleep Disturbance and Sleep-Related Impairment.^30^ Sleep Disturbance evaluates self-reported sleep quality and restoration, whereas Sleep-Related Impairment measures the impact of poor sleep on daytime alertness and function. PROMIS scores are reported as *t*-scores, with higher values indicating greater symptomatic burden.

To evaluate the robustness of findings, sensitivity analyses were conducted with two alternative measures of cognitive complaints: the Multiple Sclerosis Neuropsychological Questionnaire (MSNQ)^31^ and the Quality of Life in Neurological Disorders Cognitive Function computerised adaptive test (Neuro-QoL-Cog),^32^ as well as the BICAMS battery in place of the cognitive composite approach. These supplementary analyses were intended to confirm whether the observed results were consistent across alternate instruments, and are reported in full in Supplementary Tables 3 and 4.

### Statistical analyses

Analyses were performed using R (version 4.2.2 for macOS; R Core Team, Vienna, Austria; https://www.r-project.org/) and JASP (version 0.17.3 for macOS; Jasp Team, Amsterdam, The Netherlands; https://jasp-stats.org/) software packages. Descriptive statistics, including means and percentages, were calculated for all variables. Outliers were defined as values exceeding *z* = ±3.29 s.d. and were Winsorised by replacing them with one greater than the next highest data point. Binary classifications were computed for objective cognitive impairment and non-cognitive symptoms for descriptive purposes and the pattern analysis. Fatigue and sleep-related variables were classified as elevated at *t* ≥ 1.5 s.d. As per the test manual, SPECTRA scores were considered elevated at *t* ≥ 2 s.d. Pattern analysis was conducted using an UpSet plot^33^ to visualise the unique neuropsychological phenotypes across patients.

Bayesian statistical methods were used to support an estimation-focused and interpretable approach to statistical inference. In contrast to frequentist null-hypothesis significance testing, Bayesian analyses estimate probability distributions for effects of interest given the observed data. This allows effect sizes and their associated uncertainty to be expressed directly, using credible intervals that describe the range of values that are most plausible given the data. A further advantage of this approach is that Bayesian methods allow evidence to be quantified both in favour of, and against, a given hypothesis. This distinction is particularly relevant in applied clinical research, where a lack of association may itself be informative and where it is important to distinguish between absence of evidence and evidence for absence. Bayesian inference therefore facilitates more nuanced interpretation of findings than dichotomous significance testing alone.

Bayesian Pearson correlations were used to examine associations between the SPECTRA cognitive concerns subscale and outcome measures, with standardised effect sizes (*r*) and 95% credible intervals reported. Separate Bayesian linear regression analyses were then conducted with the Bayesian adaptive sampling algorithm in JASP to examine associations between cognitive complaints and individual objective cognitive and non-cognitive predictors. A null model was first specified to include age, education and gender. For each cognitive composite and non-cognitive symptom, an alternative model was specified by adding the predictor of interest to the null model. Each predictor was therefore evaluated in a separate model adjusting only for demographic variables, rather than within a single multivariable model, because of anticipated multicollinearity among psychometric measures and sample size considerations. The Jeffreys–Zellner–Siow prior was applied to model coefficients with an *r* scale of 0.354, and a uniform prior was assigned to each model. To ensure reproducibility, the random seed was fixed at 1, which stabilises the set of sampled models used in posterior estimation. Evidence for or against the predictive utility of each variable was assessed with the Bayes factor (BF). A *BF*_10_ > 3 was interpreted as evidence for the alternative hypothesis (i.e. variable of interest is a significant predictor of cognitive concerns), whereas a *BF*_10_ < 1/3 was considered evidence for the null hypothesis (i.e. variable of interest is not a significant predictor of cognitive concerns).^34^ Change in explained variance (Δ*R* ^2^) was used to quantify the increase in variance explained when each predictor was added to the demographic-adjusted null model. In line with Bayesian principles, no correction for multiple comparisons was applied, as Bayes factors quantify evidence for each hypothesis individually without inflating type 1 error rates.^35^

## Results

### Patient characteristics

A total of 51 patients met inclusion criteria. The majority of patients were women (*n* = 37, 73%), with a mean EDSS score of 2.4 (s.d. = 1.91), indicating mild to moderate disability. Most patients were diagnosed with RRMS (*n* = 48, 94%), with fewer cases of primary progressive multiple sclerosis (*n* = 2, 4%) and secondary progressive multiple sclerosis (*n* = 1, 2%). The most common disease-modifying therapy was ocrelizumab (*n* = 19, 37%), followed by natalizumab (*n* = 13, 25%), cladribine (*n* = 9, 18%) and others (*n* = 5, 10%). Five patients (10%) were not receiving disease-modifying therapies. Most patients were referred on the basis of self-reported cognitive complaints (*n* = 46, 90%), with fewer referrals for baseline assessment (*n* = 4, 8%) or as a result of concern raised by the primary treating neurologist (*n* = 1, 2%).

Mean *z*-scores for all cognitive domains and individual subtests were within normal limits at the group level (i.e. not more than 1.5 s.d. below the normative mean), indicating the sample was not severely impaired overall. Individual variability, however, was evident. SPECTRA cognitive concerns were severely elevated, with a mean *t*-score of 72.29 (s.d. = 16.83). Depression *t*-scores averaged 64.08 (s.d. = 14.45), indicating moderate elevation within the sample, whereas the GPI *t*-score averaged 57.49 (s.d. = 9.84), suggesting mild overall elevation. Full patient characteristics are summarised in Table 1 and Supplementary Table 2.

**Fig. 1:**
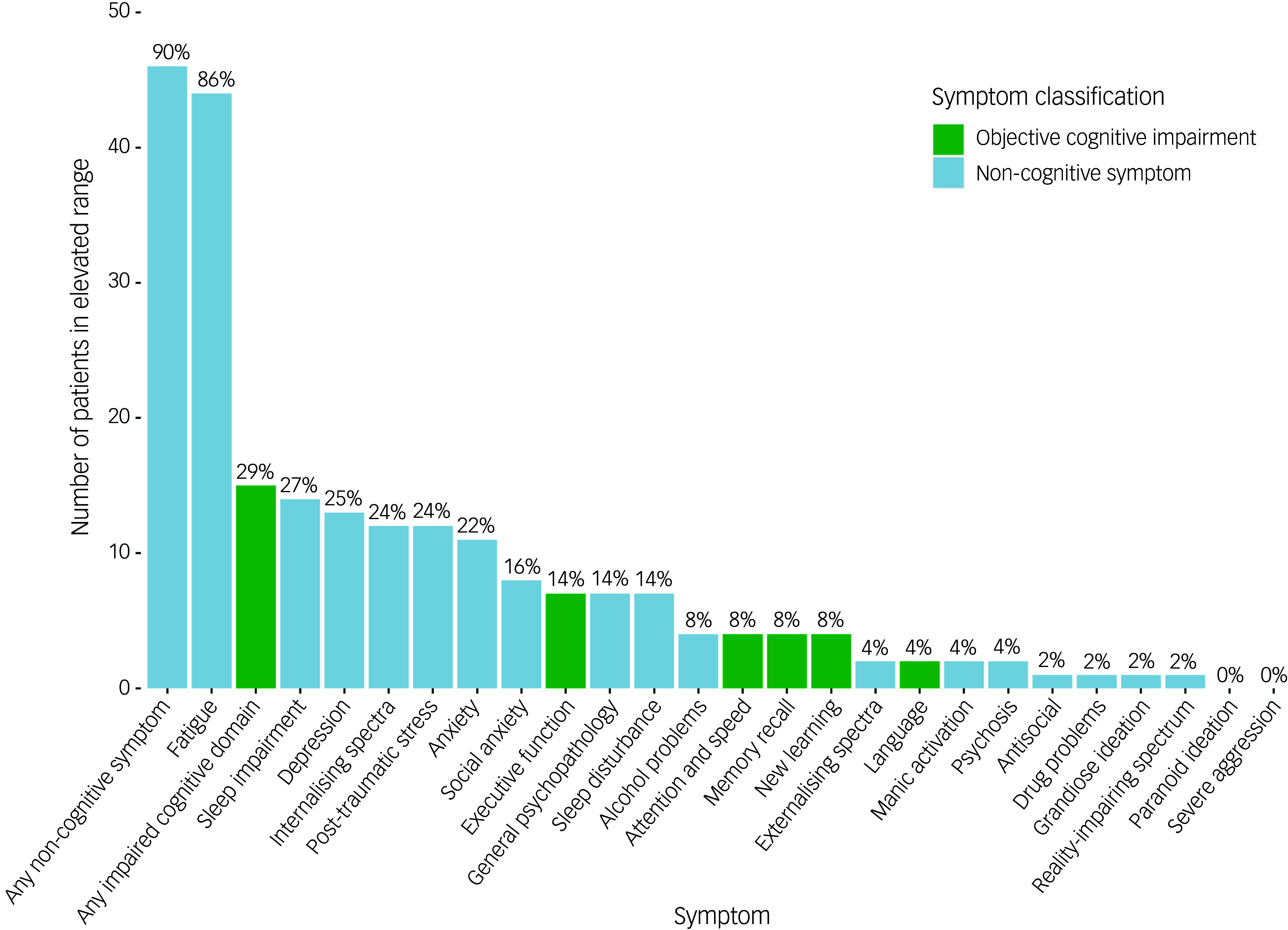
Frequency of patients with symptom elevations across objective cognitive domains and non-cognitive symptoms. Bars represent the number of patients exceeding the clinical threshold for each term.

Cognitive complaints were identified in 50 patients (98%), based on neuropsychologist classification during the clinical consultation. This high prevalence is consistent with the referral context of the clinic, where patients are commonly referred because of cognitive complaints. Given the highly skewed distribution, the binary classification of clinician-derived cognitive complaint was used only for descriptive purposes. As shown in Fig. 1, objective cognitive impairment was observed in 15 patients (29%), whereas 46 (90%) showed elevations in at least one non-cognitive symptom. Objective cognitive impairment was most frequently observed in executive function (*n* = 7, 14%). The most frequently observed non-cognitive symptom was fatigue (*n* = 44, 86%), followed by sleep impairment (*n* = 14, 28%) and depression (*n* = 13, 26%).

**Fig. 2:**
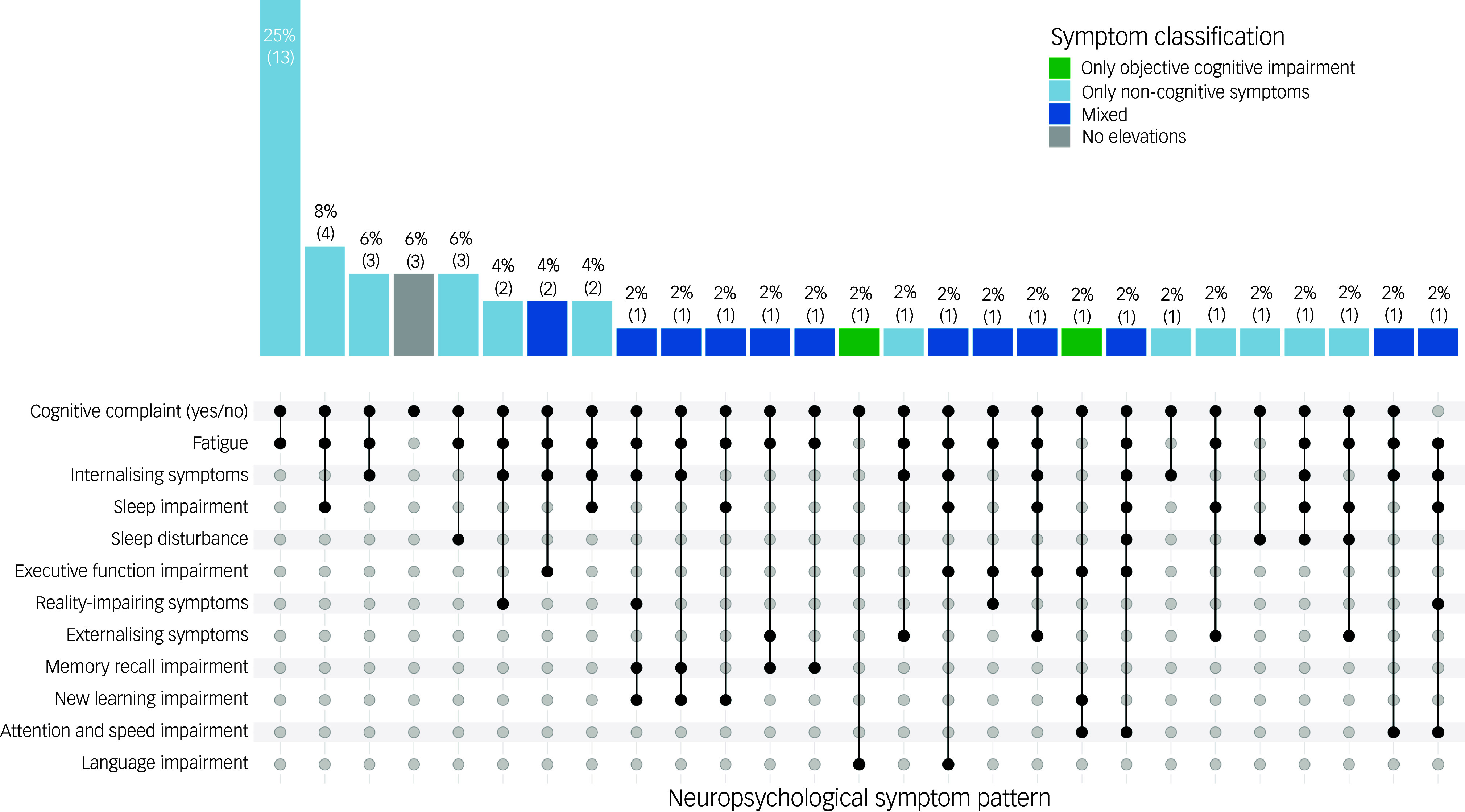
UpSet plot depicting the distribution of neuropsychological phenotypes based on objective cognitive impairment and elevations in non-cognitive symptoms. Each vertical bar represents the number of patients with a unique combination of symptoms, with absolute counts and percentages displayed above. The matrix below indicates which symptoms are present in each combination, with filled dots denoting inclusion.

As shown in Fig. 2, the pattern of cognitive and non-cognitive symptoms was highly variable across the sample. The most common overall profile was cognitive complaints without objective cognitive impairment but with at least one elevated non-cognitive symptom (*n* = 33, 65%). The most frequent specific symptom combination involved cognitive complaints with elevated fatigue and no objective cognitive impairment (*n* = 13, 25%), followed by combinations that included fatigue with either sleep disturbance (*n* = 4, 8%), internalising symptoms (*n* = 3, 6%), or both fatigue and sleep disturbance (*n* = 3, 6%) – all occurring in the absence of objective cognitive impairment.

### Predictors of cognitive complaints

**Fig. 3:**
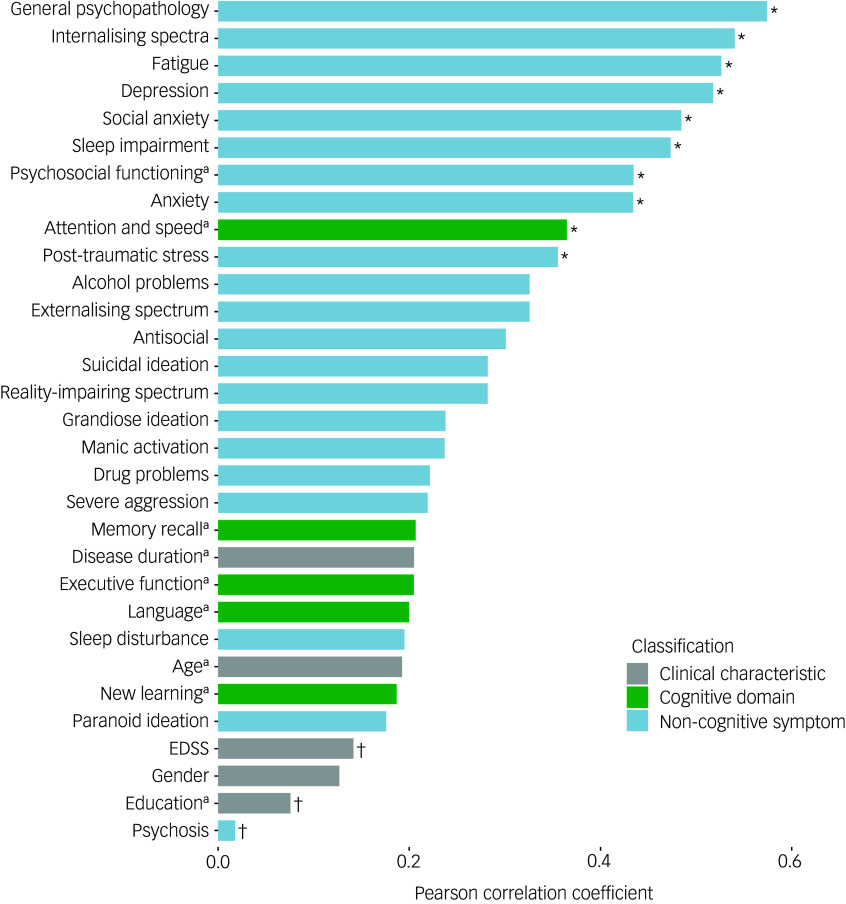
Pearson correlation between SPECTRA cognitive concerns and clinical characteristics, objective cognitive performance and non-cognitive symptoms. Bars represent the magnitude of the correlation for each variable. EDSS, Expanded Disability Status Scale. a. Absolute correlation to demonstrate the magnitude of the effect. *Bayes factor >3. ^†^Bayes factor <1/3.

As seen in Table 1, Fig. 3 and Supplementary Fig. 1, SPECTRA cognitive concerns were moderately correlated with the attention and processing speed composite (*r* = −0.37, *BF*_10_ = 5.06). Among non-cognitive symptoms, general psychopathology, internalising symptoms and fatigue showed the strongest associations with cognitive complaints (*r* = 0.52 to 0.57, *BF*_10_ > 360.00). Moderate correlations were also observed with sleep impairment (*r* = 0.47, *BF*_10_ = 69.27) and with several specific symptoms that fall within the internalising spectrum, including depression (*r* = 0.52, *BF*_10_ = 275.66), social anxiety (*r* = 0.49, *BF*_10_ = 96.54) and anxiety (*r* = 0.43, *BF*_10_ = 24.07). Lower psychosocial functioning was also associated with greater SPECTRA cognitive concerns (*r* = −0.44, *BF*_10_ = 24.33).

A Bayesian model averaging approach was used to examine associations between SPECTRA cognitive concerns and objective cognitive and non-cognitive symptoms, with each predictor entered in a separate model adjusting for gender, age and education. As shown in Table 2, overall psychopathology (GPI) showed the strongest association with cognitive complaints (*β* = 0.81, *BF*_10_ = 651.97). Fatigue, sleep impairment and internalising psychopathology were also strongly associated with SPECTRA cognitive concerns scores, each associated with a 22–26% increase in explained variance relative to the null model (*β* = 0.46–0.65, *BF*_10_ = 65.29–164.67). Within the internalising spectrum, symptoms of depression (*β* = 0.49, *BF*_10_ = 191.19) and social anxiety (*β* = 0.42, *BF*_10_ = 35.44) also emerged as relevant predictors. Several other models, including the attention and processing speed composite, produced Bayes factors above 3, but credible intervals for these predictors included zero. These effects were therefore interpreted cautiously given the uncertainty in their estimated magnitude.

**Table 2:** Separate Bayesian regression estimates of objective cognitive performance and non-cognitive symptoms as predictors of SPECTRA cognitive concerns

Variable	*β* [95% CI]	*BF*_10_	Δ*R* ^2^
Cognitive domain			
Attention and processing speed mean	−4.26 [−10.28 to 0.03]	3.54^a^	0.10
Executive function mean	−1.54 [−9.18 to 2.45]	0.80	0.03
New learning mean	−0.71 [−4.80 to 1.66]	0.71	0.08
Memory recall mean	−0.96 [−5.51 to 1.38]	0.82	0.03
Language mean	−0.92 [−6.23 to 2.17]	0.71	0.08
Non-cognitive symptom			
Sleep disturbance	0.27 [−0.05 to 0.85]	1.99	0.07
Sleep impairment	0.65 [0.23−1.06]^b^	65.29^a^	0.22
Fatigue	0.46 [0.20−0.69]^b^	148.80^a^	0.25
SPECTRA general psychopathology index	0.81 [0.42−1.18]^b^	651.97^a^	0.30
Internalising spectrum	0.54 [0.24−0.82]^b^	164.67^a^	0.26
Depression	0.49 [0.22−0.74]^b^	191.19^a^	0.26
Anxiety	0.35 [0.00−0.58]	16.92^a^	0.17
Social anxiety	0.42 [0.11−0.73]^b^	35.44^a^	0.20
Post-traumatic stress	0.26 [−0.00 to 0.56]	4.88^a^	0.12
Externalising spectrum	0.21 [−0.01 to 0.66]	2.60	0.09
Alcohol problems	0.15 [−0.00 to 0.38]	2.96	0.09
Severe aggression	0.13 [−0.12 to 0.70]	0.97	0.04
Antisocial	0.28 [−0.03 to 0.80]	2.37	0.08
Drug problems	0.15 [−0.15 to 0.80]	0.88	0.03
Reality-impairing spectrum	0.29 [−0.07 to 0.93]	1.82	0.07
Psychosis	0.01 [−0.35 to 0.32]	0.50	0.06
Paranoid ideation	0.11 [−0.13 to 0.64]	0.85	0.03
Manic activation	0.17 [−0.07 to 0.67]	1.37	0.05
Grandiose ideation	0.23 [−0.09 to 0.90]	1.38	0.05
Psychosocial functioning	−0.54 [−0.92 to 0.00]	17.73^a^	0.17
Suicidal ideation	0.15 [−0.03 to 0.47]	1.93	0.07

Δ*R* ^2^ reflects the change in explained variance associated with adding each predictor to the null model (including age, gender and education) and does not account for the effects of other cognitive or non-cognitive predictors. *β,* standardised model coefficient (mean of posterior distribution); *BF*_10_, Bayes factor for the alternative hypothesis; Δ*R* ^2^, change in coefficient of determination.

a. Bayes factor >3.

b. 0 is not included as a plausible value at the 95% level.

### Sensitivity analyses

Results were consistent across alternative measures of cognitive complaints. The SPECTRA cognitive concerns subscale showed similar patterns of association with clinical characteristics, objective cognitive impairment and non-cognitive symptoms as the MSNQ and Neuro-QoL-Cog, though effect sizes were consistently larger for the SPECTRA measure (see Supplementary Tables 3 and 4). Analyses using the BICAMS in place of the cognitive composite approach also yielded comparable results. A linear regression adjusted for age, gender and education did not strongly support either the null or the alternative hypothesis for the association between BICAMS performance and SPECTRA cognitive concerns (*β* = −2.88, 95% CI −8.64 to 0.04; *BF*_10_ = 2.30; see Supplementary Table 3).

## Discussion

This cross-sectional study used real-world data from a specialist cognitive clinic to explore the clinical utility of cognitive complaints in patients with multiple sclerosis. Given that 98% of patients reported cognitive complaints, binary classification of concerns lacked clinical relevance in this setting. Cognitive complaints defined using a severity measure provided meaningful insights about patient status, beyond objective cognitive performance. Notably, 65% of patients had no objective cognitive impairment, but exhibited clinical elevations in at least one non-cognitive symptom, most commonly fatigue, sleep impairment and internalising psychopathology. Severity of cognitive complaints independently predicted fatigue, sleep-related functional impairment and overall psychopathology, including internalising symptoms of depression and social anxiety.

Although a binary classification of cognitive complaint may reflect how many clinicians make practical decisions, this approach was not informative in our sample. Nearly all patients were classified as having cognitive complaints based on neuropsychologist impression, which limited the utility of the binary variable for distinguishing between clinical presentations. This suggests that in specialist cognitive clinics, where referrals are often based on cognitive complaint, the binary presence or absence of concern adds little beyond confirming the referral basis. In contrast, severity-based self-report measures provided a dimensional index of the extent to which patients experienced cognitive difficulties, which was reflected in graded associations with fatigue, sleep impairment and psychopathology that are directly relevant to patient care.

The strong associations between cognitive complaints and fatigue, sleep and psychopathology are consistent with previous research in multiple sclerosis populations.^10–20^ This study extends prior findings by being the first, to our knowledge, to simultaneously examine fatigue, self-reported sleep quality and a broad spectrum of psychopathology symptoms within a single cohort referred for specialist cognitive evaluation. By integrating these non-cognitive symptoms alongside objective cognitive performance, the findings offer a more complete picture of the variables associated with cognitive complaint. Notably, general psychopathology and fatigue showed the largest increases in explained variance when added individually to the demographic-adjusted null model, reinforcing the view that cognitive complaints often reflect broader intrapersonal functioning. Sensitivity analyses using alternative measures of cognitive complaint supported the robustness of these associations.

The findings are also consistent with previous research in both multiple sclerosis and broader clinical populations, where cognitive complaints often show weak correspondence with objective cognitive performance.^6,9^ Although a moderate correlation was observed between cognitive complaint severity and attention and processing speed, Bayesian regression did not support the unique contribution of objective cognitive performance when accounting for age, education and gender. This suggests that objective cognitive impairment may relate to cognitive complaints, but the nature and strength of this relationship remain unclear. One possibility is that standardised neuropsychological testing may fail to capture aspects of cognitive dysfunction that are most salient to patients in everyday, real-world contexts.^36^ Another consideration relates to how cognitive complaints are measured. Standardised questionnaires and binary clinical ratings may not adequately capture the complexity of cognitive dysfunction as it is experienced by patients. Research in Alzheimer’s disease and epilepsy has demonstrated that phenomenological typologies of cognitive complaint can distinguish between clinical groups more effectively than severity ratings alone.^37,38^ Future research should explore methods that account for the multidimensional nature of cognitive complaint in multiple sclerosis, rather than relying solely on simplified rating scales, to better characterise the relationship between subjective and objective impairment.

The mechanisms underlying the close relationship between cognitive complaints and non-cognitive symptoms in multiple sclerosis are likely to be complex and bidirectional.^36^ Non-cognitive symptoms such as fatigue, sleep-related difficulties, depression and anxiety may heighten awareness of everyday cognitive inefficiencies or reduce perceived cognitive capacity, thereby amplifying cognitive complaints. Conversely, experiencing persistent cognitive difficulties may itself contribute to emotional distress, worry or sleep disruption, particularly when cognitive changes are perceived as threatening or progressive. Evidence from broader clinical settings supports this reciprocal model.^39^ In specialist memory clinic populations, individuals presenting with cognitive complaints in the absence of objective cognitive impairment frequently exhibit a similar cluster of non-cognitive symptoms, including poor sleep, excessive fatigue, depression and anxiety,^40^ with cross-sectional and longitudinal studies suggesting potential bidirectional relationships between cognitive complaints and affective symptoms.^41^ Although causal pathways cannot be determined in the present cross-sectional study, the current findings are consistent with models in which cognitive complaints reflect an interaction between perceived cognitive change and broader intrapersonal functioning, rather than objective cognitive impairment alone.

Interpretation of objective cognitive performance in isolation of complaint findings in this cohort also warrant consideration. Executive function emerged as the most frequently impaired cognitive domain which is somewhat atypical given that slowed information processing speed is more commonly emphasised in multiple sclerosis research.^42^ Increasing evidence suggests, however, that cognitive impairment in multiple sclerosis is heterogeneous, with executive dysfunction recognised as a prominent feature in some cognitive phenotypes, particularly among individuals with more severe impairment profiles.^23^ In addition, executive function may be insufficiently characterised in much of the multiple sclerosis literature, as commonly used screening batteries do not routinely include comprehensive measures of this domain. Although executive function deficits may be associated with reduced insight in neurological populations, a further consideration relates to how executive function is examined. Many executive measures incorporate a timed component and place substantial demands on processing speed, such that apparent executive impairment may reflect broader cognitive inefficiency rather than isolated executive dysfunction. In this context, executive impairment would not necessarily be expected to preclude awareness of cognitive difficulty, which is consistent with the high prevalence of cognitive complaints observed in this cohort.

A key consideration in this study is the nature of the sample, which comprised patients referred specifically for neuropsychological evaluation within a tertiary neuroimmunology service. High rates of cognitive complaint were expected, given that referrals were typically prompted following patient-reported difficulties. Although this approach may limit the generalisability of findings to the broader multiple sclerosis population, it strengthens the study’s clinical relevance by ensuring that the results apply directly to the patient group most likely to seek or be referred for cognitive opinion. Given that 98% of the sample reported cognitive complaints, these findings contribute to a better understanding of the symptoms associated with subjective cognitive dysfunction in clinical settings. The cross-sectional design, however, precludes conclusions about the temporal course of cognitive complaints or their relationship to subsequent objective cognitive decline. Longitudinal studies are needed to determine whether cognitive complaints in multiple sclerosis predict later objective cognitive impairment, or whether they remit alongside improvements in modifiable non-cognitive symptoms. Future research could further extend these findings by examining cognitive complaints in a broader, community-based multiple sclerosis population.

A further limitation concerns the modelling approach used to examine predictors of cognitive complaints. In this modestly sized clinical sample, predictors were examined in separate models adjusting for demographic variables only (i.e. age, gender and education). Consequently, the relative contribution of individual cognitive and non-cognitive symptoms was not evaluated within a single multivariable framework. Given the substantial conceptual and empirical overlap between non-cognitive symptoms such as fatigue, sleep-related difficulties and mood disturbance, future studies with larger samples will be better positioned to apply multivariable or hierarchical modelling approaches to characterise shared versus unique effects and formally examine potential mediation pathways.

The current findings highlight the need to prioritise cognitive complaints in multiple sclerosis as a clinically meaningful signal for intervention (see Box 2 for the key clinical implications for multiple sclerosis clinicians). Although objective cognitive impairment was most often absent, the most common presentation among patients referred for cognitive evaluation involved cognitive complaints alongside elevated non-cognitive symptoms. Rather than dismissing this discrepancy as a lack of insight on the part of the patient,^43^ cognitive complaints in multiple sclerosis may serve as an important indicator for investigating and managing potentially modifiable non-cognitive symptoms. Perceived cognitive dysfunction, even in the absence of objective impairment, can lead to significant distress and functional disability, which may persist for months to years without appropriate intervention.^44,45^ Evidence from multiple sclerosis populations suggests that addressing non-cognitive symptoms such as depression and fatigue can improve subjective perceptions of cognition, potentially mitigating long-term negative outcomes.^14,46^ Recognising and responding to cognitive complaints, regardless of whether objective impairment is present, may therefore be a crucial step in optimising patient care.

Box 2 Clinical take-home message

In this specialist multiple sclerosis cognitive clinic cohort, cognitive complaints were almost universal, but objective cognitive impairment was present in fewer than a third of patients.

Treating cognitive complaint as a binary clinical marker was therefore not useful. Severity of concerns, however, tracked most strongly with fatigue, sleep-related impairment and overall psychopathological burden.

When a patient with multiple sclerosis reports cognitive difficulties, early identification and management of fatigue, sleep disturbance and mood/anxiety may represent a pragmatic first step while awaiting, or in parallel with, neuropsychological assessment.

In conclusion, cognitive complaints in multiple sclerosis may signal clinically significant non-cognitive symptoms, including psychopathology, fatigue and sleep dysfunction. These findings reinforce the value of interpreting complaints not only in relation to neuropsychological test performance, but also as potential indicators of broader clinical needs that can subsequently be targeted for intervention. Future research should further explore how best to capture and respond to cognitive complaints within multidisciplinary multiple sclerosis care.

## Supporting information

10.1192/bjo.2026.11041.sm001 Roberts et al. supplementary material Roberts et al. supplementary material

## Data Availability

Materials and de-identified data can be provided to suitably qualified researchers by contacting the corresponding author, S.R.

## Supplementary material

The supplementary material is available online at https://doi.org/10.1192/bjo.2026.11041
