# Supplementary material for: The relationship between cognitive complaints and burden of non-cognitive symptoms in multiple sclerosis
Source: BJPsych Open. 2026 May 6;12(3):e127. doi: 10.1192/bjo.2026.11041 (PMC13150722; doi:10.1192/bjo.2026.11041)
Supplement: Roberts et al. supplementary material [file S2056472426110412sup001.docx]

**Supplementary material**

**Supplementary Table 1**. Description of measures comprising each cognitive composite.

| **Cognitive measure** | | **Description** | **Norms** |
| --- | --- | --- | --- |
| Attention and processing speed | | |  |
|  | SDMT – oral version [1]# | Patients are presented with a key pairing symbols with digits and are asked to verbally match as many symbols to their corresponding digits as possible within 90 seconds. | Test manual |
|  | TMT Part A [2] | Patients draw a line connecting 25 numbered circles distributed randomly on a page in ascending order as quickly as possible. Completion time is recorded. | [3] |
|  | WAIS-IV Digit Span Forward [4] | Patients are read sequences of digits and asked to repeat them in the same order. Sequence length increases across trials. | Test manual |
| Executive function | |  |  |
|  | COWAT orthographic lexical retrieval [5] | Patients are asked to produce as many words as possible beginning with a given letter (i.e., F, A, S) within 60 seconds per letter. | [5] |
|  | TMT Part B [2] | Patients are asked to draw a line alternating between numbers and letters in ascending sequence (e.g., 1-A-2-B). The circles are randomly distributed on the page, and completion time for each is recorded. | [5] |
|  | Victoria Stroop Test [5] | The test includes three conditions, each with 24 items: naming the colours of dots, naming the colour of neutral words, and naming the ink colour of colour words printed in incongruent colours. An interference score was calculated as the time to complete the dot condition divided by the time to complete the incongruent condition. | [5] |
|  | WAIS Digit Span Backward [4] | Patients are read sequences of digits and asked to repeat them in reverse order. Sequence length increases across trials. | Test manual |
| New learning | |  |  |
|  | BVMT-R total recall [6]# | Patients are shown a 2x3 array of abstract designs for 10 seconds and then asked to draw the designs from memory. This process is repeated across three learning trials. The total recall score reflects cumulative recall across the three trials. | Test manual |
|  | CVLT-II List A total trials 1-5 [7]# | Patients are read a 16-item word list composed of four semantic categories over five learning trials and asked to recall as many words as possible after each presentation. | Test manual |
| Memory | |  |  |
|  | BVMT-R delayed recall [6] | Patients are asked to draw the previously viewed 2x3 array of abstract designs from memory after a 25-minute delay. | Test manual |
|  | CVLT-II long delay [7] | Patients are asked to recall as many items as possible from a previously learned 16-item word list after a 30-minute delay. | Test manual |
| Language | |  |  |
|  | BNT [8] | A 60-item measure of visual confrontation naming. Patients are presented with black-and-white line drawings of objects arranged in order of increasingly difficulty, ranging from high- to low-frequency vocabulary. If a spontaneous response is not produced, semantic or phonemic cues may be provided. | [9] |
|  | COWAT semantic lexical retrieval [9] | Patients are asked to name as many words as possible belonging to a specified semantic category (i.e., animals) in 60 seconds. | [5] |

Note: # = Subtest of BICAMS. Abbreviations: BNT = Boston Naming Test; BVMT-R = Brief Visuospatial Memory Test-Revised; COWAT = Controlled Oral Word Association Test; CVLT-II = California Verbal Learning Test-Second Edition; Norms = normative data used for standardized score calculation. SDMT = Symbol Digit Modalities Test, TMT = Trail Making Test; WAIS-IV = Wechsler Adult Intelligence Scale-Fourth Edition.

References

1. Smith A. *Symbol Digit Modalities Test (SDMT) Manual (revised)*. Western Psychological Services, 1982.

2. Reitan RM. The relation of the Trail Making Test to organic brain damage. *J Consult Psychol* 1955; 19: 393–394.

3. Tombaugh TN. Trail Making Test A and B: Normative data stratified by age and education. *Arch Clin Neuropsychol* 2004; 19: 203–214.

4. Wechsler D. *Wechsler Adult Intelligence Scale - Fourth Edition (WAIS-IV): Australian and New Zealand Language Adapted Edition*. The Psychological Corporation, 2008.

5. Strauss E, Sherman EMS, Spreen O. *A compendium of neuropsychological tests: Administration, norms, and commentary, 3rd ed*. New York, NY, US: Oxford University Press, 2006.

6. Benedict RHB, Schretlen D, Groninger L, et al. Revision of the Brief Visuospatial Memory Test: Studies of normal performance, reliability, and validity. *Psychol Assess* 1996; 8: 145–153.

7. Delis DC, Kramer JH, Kaplan E, Ober BA. *The California Verbal Learning Test – second edition. Adult version. Manual.* Psychological Corporation, 2000.

8. Kaplan E, Goodglass H, Weintraub S. *The Boston Naming Test*. Lea & Febiger, 1983.

9. Tombaugh TN, Hubley AM. The 60-item Boston Naming Test: Norms for cognitively intact adults aged 25 to 88 years. *J Clin Exp Neuropsychol* 1997; 19: 922–932.

**Supplementary Table 2.** Cognitive composite subtest descriptives and correlations with severity of cognitive concerns (SPECTRA)

| **Cognitive measure** | | **Mean (SD)** | **Pearson’s *r* [95%CI]** | ***BF_10_*** |
| --- | --- | --- | --- | --- |
| Attention & processing speed composite | |  |  |  |
|  | Digit Span forward | -0.09 (0.80) | -0.04 [-0.31, 0.23] | 0.18^†^ |
|  | Symbol Digit Modalities Test | -0.43 (0.07) | -0.46 [-0.64, -0.20] | 41.16* |
|  | Trail Making Test part-A | 0.14 (1.06) | -0.30 [-0.52, -0.02] | 1.53 |
| Executive function composite | |  |  |  |
|  | Orthographic lexical retrieval | -0.79 (1.04) | 0.14 [-0.14,0.39] | 0.28^†^ |
|  | Stroop C/D | 0.43 (0.80) | -0.02 [-0.28, 0.25] | 0.18^†^ |
|  | Digit Span backward | -0.18 (0.79) | -0.13 [-0.38, 0.15] | 0.26^†^ |
|  | Trail Making Test part-B | -0.68 (1.45) | -0.36 [-0.57, -0.09] | 5.00* |
| New learning composite | |  |  |  |
|  | BVMT-R total recall | -0.19 (1.20) | -0.18 [-0.43, 0.10] | 0.39 |
|  | CVLT-II list A total T1-5 | 0.26 (1.23) | -0.15 [-0.40, 0.13] | 0.30^†^ |
| Memory recall composite | |  |  |  |
|  | BVMT-R delayed recall | 0.15 (1.06) | -0.09 [-0.35, 0.19] | 0.21^†^ |
|  | CVLT-II long delay | 0.05 (1.25) | -0.25 [-0.48, 0.03] | 0.82 |
| Language | |  |  |  |
|  | Boston Naming Test | -0.41 (1.15) | -0.12 [-0.37, 0.16] | 0.24^†^ |
|  | Semantic lexical retrieval | 0.17 (1.09) | -0.18 [-0.42, 0.10] | 0.37 |
| BICAMS battery | | -0.12 (0.93) | -0.32 [-0.54, -0.05] | 2.23 |

Note: Cognitive terms represent z-scores. Abbreviations: *BF_10_* = Bayes factor for the alternative hypothesis; BICAMS = Brief International Cognitive Assessment for MS; BVMT-R = Brief Visuospatial Memory Test revised; CVLT-II = California Verbal Learning Test second edition. ^†^ = Bayes factor < 1/3. * = Bayes factor > 3.

**Supplementary Table 3.** Sensitivity analysis – Correlations with MSNQ and Neuro-QoL-Cog cognitive complaint

|  | | | **MSNQ** | | **Neuro-QoL-Cog^a^** | |
| --- | --- | --- | --- | --- | --- | --- |
|  | | | **Pearson’s *r* [95%CI]** | ***BF_10_*** | **Pearson’s *r* [95%CI]** | ***BF_10_*** |
| **Clinical characteristic** | | |  |  |  |  |
| Age, years | | | -0.03 [-0.29, 0.25] | 0.18^†^ | -0.03 [-0.29, 0.25] | 0.18^†^ |
| Education, years | | | -0.07 [-0.33, 0.2] | 0.20^†^ | 0.04 [-0.23, 0.3] | 0.18^†^ |
| Disease duration, years | | | -0.18 [-0.42, 0.1] | 0.37 | 0.29 [0.01, 0.51] | 1.31 |
| EDSS | | | 0.11 [-0.18, 0.37] | 0.24^†^ | -0.04 [-0.31, 0.24] | 0.19^†^ |
| **Cognitive domain** | | |  |  |  |  |
| Attention & processing speed | | | -0.13 [-0.38, 0.15] | 0.26^†^ | 0.10 [-0.17, 0.36] | 0.22^†^ |
| Executive function | | | -0.02 [-0.29, 0.25] | 0.18^†^ | -0.06 [-0.32, 0.21] | 0.19^†^ |
| New learning | | | -0.13 [-0.38, 0.15] | 0.26^†^ | 0.09 [-0.19, 0.35] | 0.21^†^ |
| Memory recall | | | -0.24 [-0.47, 0.04] | 0.72 | 0.28 [0.00, 0.5] | 1.16 |
| Language | | | 0.14 [-0.14, 0.39] | 0.28^†^ | -0.04 [-0.3, 0.23] | 0.18^†^ |
| BICAMS battery | | | -0.21 [-0.45, 0.07] | 0.49 | 0.15 [-0.13, 0.40] | 0.29^†^ |
| **Non-cognitive symptom** | | |  |  |  |  |
| Sleep disturbance | | | 0.34 [0.06, 0.55] | 3.02* | -0.22 [-0.46, 0.06] | 0.55 |
| Sleep impairment | | | 0.42 [0.15, 0.61] | 16.31* | -0.43 [-0.62, -0.16] | 19.17* |
| Fatigue | | | 0.53 [0.29, 0.69] | 437.24* | -0.6 [-0.75, -0.38] | 7848.62* |
| SPECTRA general psychopathology index | | | 0.44 [0.18, 0.63] | 26.11* | -0.45 [-0.63, -0.19] | 31.53* |
|  | Internalizing spectrum | | 0.4 [0.13, 0.6] | 10.79* | -0.5 [-0.67, -0.24] | 133.15* |
|  |  | Depression | 0.4 [0.13, 0.6] | 10.20* | -0.54 [-0.7, -0.3] | 616.30* |
|  |  | Anxiety | 0.36 [0.09, 0.57] | 4.39* | -0.36 [-0.57, -0.09] | 4.88* |
|  |  | Social anxiety | 0.36 [0.08, 0.56] | 4.17* | -0.48 [-0.65, -0.22] | 73.73* |
|  |  | Post-traumatic stress | 0.23 [-0.05, 0.46] | 0.61 | -0.34 [-0.55, -0.07] | 3.08* |
|  | Externalizing spectrum | | 0.25 [-0.03, 0.48] | 0.83 | 0.04 [-0.23, 0.31] | 0.18^†^ |
|  |  | Alcohol problems | 0.23 [-0.05, 0.46] | 0.61 | -0.05 [-0.31, 0.23] | 0.18^†^ |
|  |  | Severe aggression | 0.16 [-0.12, 0.4] | 0.31^†^ | 0.03 [-0.24, 0.3] | 0.18^†^ |
|  |  | Antisocial | 0.2 [-0.08, 0.44] | 0.48 | 0.05 [-0.22, 0.31] | 0.19^†^ |
|  |  | Drug problems | 0.29 [0.02, 0.52] | 1.45 | 0.04 [-0.24, 0.3] | 0.18^†^ |
|  | Reality-impairing spectrum | | 0.11 [-0.17, 0.36] | 0.23^†^ | -0.12 [-0.37, 0.16] | 0.24^†^ |
|  |  | Psychosis | -0.02 [-0.29, 0.25] | 0.18^†^ | -0.07 [-0.33, 0.21] | 0.19^†^ |
|  |  | Paranoid ideation | 0.05 [-0.22, 0.31] | 0.19^†^ | 0.07 [-0.2, 0.33] | 0.20^†^ |
|  |  | Manic activation | 0.04 [-0.23, 0.31] | 0.18^†^ | -0.12 [-0.37, 0.16] | 0.24^†^ |
|  |  | Grandiose ideation | 0.17 [-0.11, 0.42] | 0.35 | -0.17 [-0.41, 0.11] | 0.34 |
|  | Psychosocial functioning | | -0.37 [-0.58, -0.1] | 5.94* | 0.45 [0.2, 0.64] | 39.95* |
|  | Suicidal ideation | | 0.12 [-0.16, 0.37] | 0.25^†^ | -0.07 [-0.33, 0.21] | 0.19^†^ |
|  | Cognitive concerns | | 0.75 [0.58, 0.84] | 35960000* | -0.6 [-0.74, -0.38] | 6927.09* |
| Neuro-QoL-Cog | | | -0.62 [-0.76, -0.4] | 16668.84* | - | - |

Abbreviations: *BF_10_* = Bayes factor for the alternative hypothesis; EDSS = Expanded Disability Status Scale; MSNQ = Multiple Sclerosis Neuropsychological Questionnaire; Neuro-QoL-Cog = Quality of Life in Neurological Disorders cognitive function scale. ^a^Lower scores on the Neuro-QoL-Cog indicate higher cognitive concerns. ^†^ = Bayes factor <1/3. * = Bayes factor > 3.

**Supplementary Table 4**. Sensitivity analysis – Separate Bayesian regression estimates for MSNQ and Neuro-QoL-Cog as predictors of objective impairment and non-cognitive factors.

|  | | | **MSNQ** | | **Neuro-QoL-Cog^a^** | |
| --- | --- | --- | --- | --- | --- | --- |
|  | | | **β [95% CI]** | ***BF_10_*** | **β [95% CI]** | ***BF_10_*** |
| **Cognitive domain** | | |  |  |  |  |
| Attention & processing speed mean | | | -0.15 [-3.35, 1.83] | 1.00 | 0.42 [-1.00, 3.15] | 0.73 |
| Executive function mean | | | 0.41 [-1.97, 4.66] | 0.60 | -0.20 [-2.91, 2.08] | 0.56 |
| New learning mean | | | -0.12 [-2.25, 1.16] | 0.55 | 0.23 [-0.72, 1.98] | 0.68 |
| Memory recall mean | | | -0.64 [-3.34, 0.71] | 0.95 | 1.23 [-0.08, 3.34] | 2.76 |
| Language mean | | | 0.55 [-1.08, 3.81] | 0.76 | -0.25 [-2.32, 1.28] | 0.62 |
| **Non-cognitive symptom** | | |  |  |  |  |
| Sleep disturbance | | | 0.24 [0.00, 0.54] | 4.29* | -0.6 [-0.32, 0.05] | 1.07 |
| Sleep impairment | | | 0.28 [0.00, 0.49] | 10.92* | -0.23 [-0.39, -0.00] | 15.62* |
| Fatigue | | | 0.27 [0.13, 0.41] ^‡^ | 235.82* | -0.25 [-0.35, -0.15] ^‡^ | 2566.57* |
| SPECTRA general psychopathology index | | | 0.40 [0.15, 0.64] ^‡^ | 81.66* | -0.24 [-0.41, 0.00] | 17.04* |
|  | Internalizing spectrum | | 0.25 [0.00, 0.40] | 27.76* | -0.20 [-0.35, -0.07] ^‡^ | 39.61* |
|  |  | Depression | 0.24 [0.06, 0.41] ^‡^ | 34.81* | -0.20 [-0.33, -0.08] ^‡^ | 74.87* |
|  |  | Anxiety | 0.22 [0.00, 0.36] | 21.98* | -0.11 [-0.24, 0.00] | 5.31* |
|  |  | Social anxiety | 0.17 [0.00, 0.33] | 7.14* | -0.19 [-0.33, -0.06] ^‡^ | 35.21* |
|  |  | Post-traumatic stress | 0.07 [-0.03, 0.27] | 1.45 | -0.09 [-0.23, 0.00] | 2.94 |
|  | Externalizing spectrum | | 0.11 [-0.04, 0.34] | 1.44 | -0.00 [-0.13, 0.11] | 0.53 |
|  |  | Alcohol problems | 0.04 [ -0.02, 0.18] | 1.32 | -0.00 [-0.09, 0.05] | 0.54 |
|  |  | Severe aggression | 0.06 [-0.10, 0.37] | 0.81 | 0.01 [-0.17, 0.17] | 0.53 |
|  |  | Antisocial | 0.06 [-0.09, 0.37] | 0.85 | -0.01 [-0.19, 0.13] | 0.53 |
|  |  | Drug problems | 0.22 [-0.01, 0.59] | 2.86 | 0.01 [-0.19, 0.19] | 0.54 |
|  | Reality-impairing spectrum | | 0.04 [-0.14, 0.38] | 0.65 | -0.03 [-0.25, 0.14] | 0.63 |
|  |  | Psychosis | 0.01 [-0.19, 0.19] | 0.53 | -0.01 [-0.19, 0.10] | 0.55 |
|  |  | Paranoid ideation | -0.00 [-0.25, 0.19] | 0.52 | 0.02 [-0.11, 0.24] | 0.59 |
|  |  | Manic activation | 0.02 [-0.11, 0.25] | 0.59 | -0.02 [-0.12, 0.12] | 0.59 |
|  |  | Grandiose ideation | 0.08 [-0.10, 0.45] | 0.88 | -0.01 [-0.38, 0.07] | 1.22 |
|  | Psychosocial functioning | | -0.30 [-0.52, 0.00] | 16.29* | 0.26 [0.00, 0.41] | 29.88* |
|  | Suicidal ideation | | 0.01 [-0.08, 0.17] | 0.58 | -0.02 [-0.14, 0.05] | 0.72 |

Note: Null model includes age, education, and sex. 95% CI = credible intervals for the model coefficient; β = standardized model coefficient (mean of posterior distribution); *BF_10_* = Bayes factor for the alternative hypothesis; MSNQ = Multiple Sclerosis Neuropsychological Questionnaire; Neuro-QoL-Cog = Quality of Life in Neurological Disorders cognitive function scale. ^a^Lower scores on the Neuro-QoL-Cog indicate greater cognitive complaints. * = Bayes factor > 3. ^‡^ = 0 is not included as a plausible value at the 95% level.


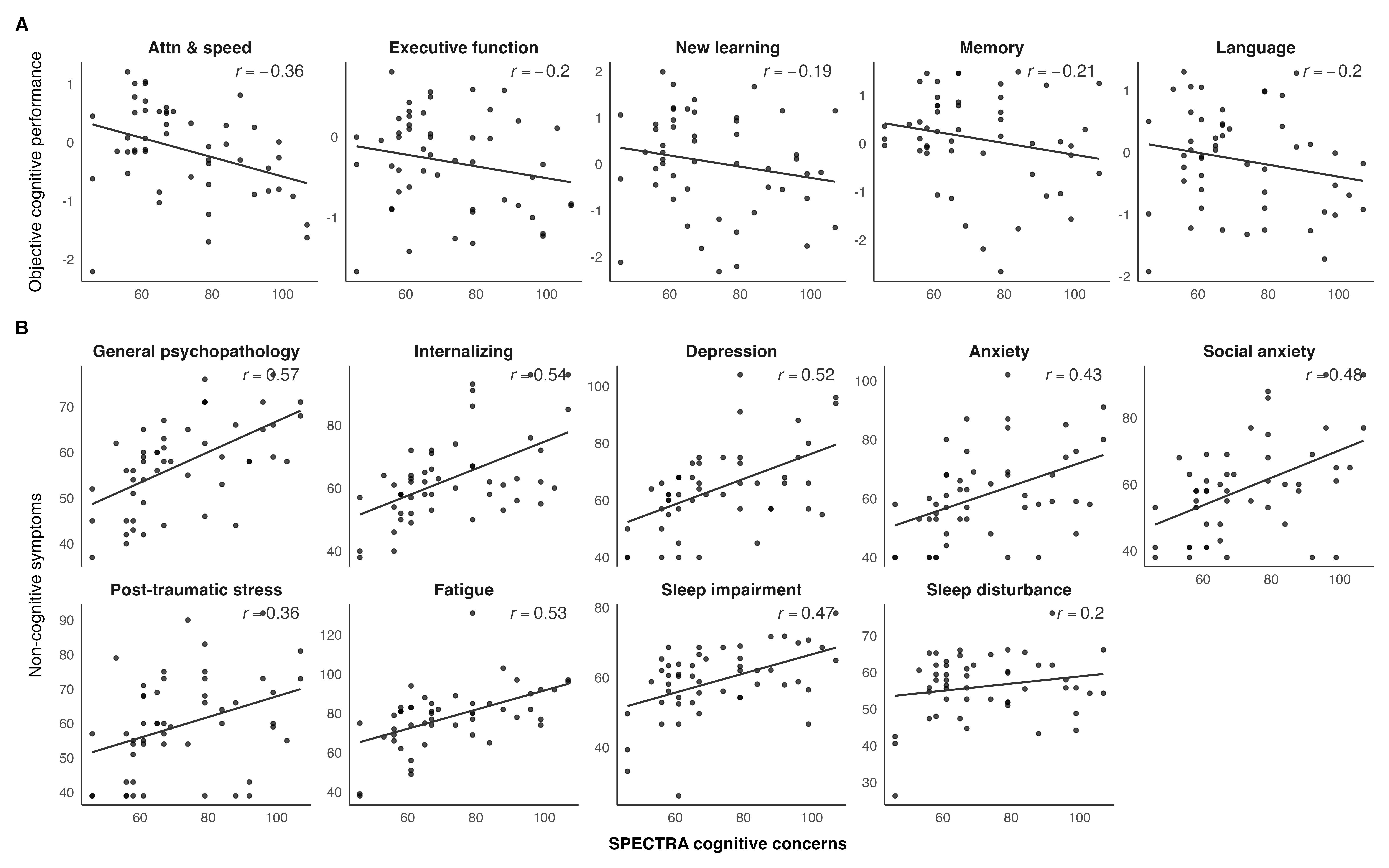


**Supplementary Fig 1.** Scatterplots showing associations between SPECTRA cognitive concerns t-scores and selected objective and non-cognitive measures in patients with multiple sclerosis. Lines represent least-squares linear regression fits, and Pearson correlation coefficients (*r*) are shown in the upper-right corner of each panel. Higher SPECTRA cognitive concerns scores indicate greater perceived cognitive difficulties. **(A)** Objective cognitive performance (composites expressed as z-scores). **(B)** Non-cognitive symptoms (scores express as t-scores).
